# Catheter ablation of idiopathic high-burden premature ventricular complexes: A case report

**DOI:** 10.1016/j.hrcr.2022.10.001

**Published:** 2022-11-15

**Authors:** Margaret Harvey

**Affiliations:** Department of Acute and Tertiary Care, College of Nursing, University of Tennessee Health Science Center, Memphis, Tennessee

**Keywords:** Premature ventricular complexes, Ventricular arrhythmias, Catheter ablation, Left ventricular outflow tract, Palpitations

## Introduction

Cardiac electrophysiology physicians routinely receive referrals to evaluate and manage patients with frequent premature ventricular complexes (PVCs). This case report describes a patient referred to the electrophysiology service for an abnormal electrocardiogram (ECG), palpitations, and dizziness.

## Case report

The patient is a 63-year-old woman with a history of hypertension*,* hyperlipidemia, and coronary artery disease with stenting to the left anterior descending artery. The patient reported frequent symptomatic palpitations occurring both at rest and with mild physical exertion. She denied any other pulmonary or cardiac symptoms and was receiving guideline-directed medical therapy in the form of aspirin, atorvastatin, omeprazole, metoprolol tartrate, and losartan. An initial cardiac workup included a 12-lead ECG that revealed normal sinus rhythm with frequent PVCs with an atypical outflow pattern, a 30-day event monitor indicating a high burden of PVCs (21.23%), and a cardiac catheterization that showed no progressive coronary artery disease. A transthoracic echocardiogram revealed a normal left ventricular ejection fraction of 55%–60% with normal regional wall motion and valvular structure and function. Based on the presenting symptoms and cardiac assessment, we recommended a ventricular catheter ablation procedure as a means of mitigating the PVCs and associated symptoms based on current guidelines and expert consensus statement for management of ventricular arrhythmias.^1^^,^^2^ The procedure was discussed with the patient, including the benefits and risks, and the patient wished to proceed. During the procedure, 3-dimensional electroanatomic mapping of the right ventricular outflow tract, left ventricular outflow tract (LVOT), and aortic cusps was performed with the CARTO system (Biosense Webster, Irvine, CA), which was then followed by radiofrequency ablation of PVCs arising from the LVOT at the aortomitral continuity. The patient tolerated the procedure well and was in stable condition postoperatively. At the 1-month follow-up appointment, the patient reported marked decrease in palpitations that was confirmed with the ECG.

## Discussion

PVCs are common and increase in frequency with age. Frequent PVCs are defined as more than 30 per hour, and those with a high burden (>15% of total number of beats) are at greater risk for developing PVC-induced cardiomyopathies.^1^ Noninvasive evaluation for patients suspected of having a high burden of PVCs should include a baseline 12-lead ECG, exercise stress treadmill testing, ambulatory cardiac monitoring, and echocardiography to assess global and regional myocardial function and valvular structure and function.^1^ This case highlights a patient with a high burden of idiopathic, outflow tract PVCs (21.23%) arising from the LVOT at the aortomitral continuity. Typically, PVCs not associated with underlying structural disease are commonly referred to as idiopathic and monomorphic in nature.^1^ Catheter ablation (class of recommendation level I) is recommended for patients with symptomatic outflow tract ventricular arrhythmias (VAs) in an otherwise normal heart where antiarrhythmic agents are ineffective, not tolerated, or not the patient’s preference.^1^ The 2019 HRS/EHRA/APHRS/LAHRS Expert Consensus Statement on Catheter Ablation of Ventricular Arrhythmias^2^ provides additional recommendations in that catheter ablation can be useful (class of recommendation level IIa) for VAs of LVOT origin. Complications from catheter ablation of outflow tract VAs are infrequent, but may include venous access site bleeding, pericardial tamponade, and damage to the coronary arteries.^1^ When counseling a patient with treatment options for high-burden PVCs, it is important to discuss the benefits and risks and patient preferences using a shared decision-making approach.^1^ In summary, this case report highlights the evaluation and management of a patient with a high burden of PVCs originating from the LVOT using catheter ablation. There has been a rapid development of VA catheter ablation over the past 10 years, and the most recent consensus statement offers additional guidance and recommendations.^1^^,^^2^
